# Prevalence of intimate partner violence and associated factors amongst women attending antenatal care at Outapi clinic, Namibia: A descriptive survey

**DOI:** 10.4102/phcfm.v9i1.1512

**Published:** 2017-12-06

**Authors:** Leonard T. Bikinesi, Robert Mash, Kate Joyner

**Affiliations:** 1 Division of Family Medicine and Primary Care, Stellenbosch University, South Africa; 2 Department of Nursing and Midwifery, Stellenbosch University, South Africa

## Abstract

**Background:**

Intimate partner violence (IPV) is a significant and largely hidden public health problem for all women and, during pregnancy, can have significant effects on the health of both mother and the unborn baby. Previous Namibian studies suggest rates of IPV as high as 36%, although few studies have been conducted in primary care.

**Aim:**

To determine the prevalence of IPV amongst women attending antenatal care.

**Setting:**

Outapi primary care clinic, Namibia.

**Methods:**

A descriptive survey administering a validated questionnaire to 386 consecutive participants.

**Results:**

The mean age of the participants was 27.5 years (standard deviation = 6.8), 335 (86.8%) were unmarried, 215 (55.7%) had only primary school education and 237 (61.4%) were in their third trimester. Overall, 51 participants (13.2%) had HIV and 44 (11.4%) had teenage pregnancies. The reported lifetime prevalence of IPV was 39 (10.1%), the 12-month prevalence was 35 (9.1%) and the prevalence during pregnancy was 31 (8.0%). Emotional abuse was the commonest type of abuse in 27 (7.0%). The commonest specific abusive behaviour was refusing to provide money to run the house or look after the children whilst the partner spent money on his priorities (4.9%). Increased maternal age was associated with an increase in the occurrence of IPV.

**Conclusion:**

The reported lifetime prevalence of IPV was 10.1%, with emotional abuse being the commonest type of abuse. Increased age was associated with an increase in reported IPV. IPV is significant enough to warrant that healthcare providers develop guidelines to assist women affected by IPV in Namibia.

## Introduction

Intimate partner violence (IPV) is widely recognised as an important public health problem, both because of the acute morbidity and mortality associated with assault and because of its longer term impact on women’s and, indirectly, children’s mental and physical health.^1^ The World Health Organization’s (WHO) multi-country study on women’s health and domestic violence concluded that 15%–71% of women experience sexual or physical violence from an intimate partner.^2^ IPV refers to behaviour by a current or ex-intimate partner, or would-be rejected lover, that causes physical, sexual or psychological harm. It is characterised by physical aggression, sexual coercion, controlling behaviour and psychological, cultural, spiritual and financial abuse.^3,4^

In Namibia, over one-third of women have reported physical or sexual violence from an intimate partner.^2^ This violence occurs amongst women of varying ages and also during pregnancy. Amongst pregnant women, between one-quarter and one-half were severely assaulted (kicked or punched in the abdomen), and in more than 90% of cases IPV was committed by the partner responsible for the pregnancy.^2^ IPV during pregnancy is important as it increases the risk of low birth weight infants, pre-term delivery, neonatal and maternal death. It impairs maternal mental health, predisposing the mother to common mental disorders such as anxiety, depression, alcohol or substance abuse. This mental suffering reduces her ability to function effectively thereby compromising bonding and breast-feeding postpartum and significantly impacting the first 1000 days of the infant’s life.^5,6^

In Namibia, the destructive effects of IPV on maternal mental health have also been found in links to depression and suicide.^7^ Physical consequences ranged from bruising, which accounted for 49% of the injuries, to more serious injuries such as stab wounds and gunshots.^7^ Violence against women results in physical, mental, sexual, reproductive and other health problems and significantly increases vulnerability to HIV.^8^

Despite the problem being highlighted in the media, little research has been conducted to determine the current prevalence and range of violence in Namibia. In Windhoek, Namibia, the lifetime prevalence of IPV amongst women was found to be 36%.^2^ Physical abuse amongst pregnant women has been reported at around 6% for Namibia and 3.8% in the Omusati region, where this research was conducted, although other types of IPV were not studied.^2,10^ Data from studies conducted in neighbouring countries reveal higher levels of IPV amongst women, for example, in Botswana a lifetime prevalence of 50% and in Zimbabwe 61%, which is amongst the highest ever recorded, whilst the South Africa rate (38%) is similar to Windhoek.^10,11,12^

In 2016, the World Health Assembly endorsed a global plan of action to strengthen the role of health systems within a national multisectoral response to address interpersonal violence, especially against women and children.^13^ To fully implement such an action, there is a great need to understand the dynamics and spectrum of violence against women in Namibia.

The primary care facilities are usually the first point of entry for people presenting with a health problem. Antenatal care provides a potentially important window of opportunity for identifying IPV. For many women in low resource settings, this will be their only contact with healthcare providers. Ideally, women will be seen four times during a pregnancy and once postpartum, and the possibility of follow-up therefore offers an ideal opportunity for addressing issues of abuse.^14^ This would support the view that all adult females in primary care practice be routinely screened for domestic violence, although others have recommended more selective case-finding.^15^ Recognising IPV is vital for any effective strategy to combat the problem. Attention to recognising IPV at primary care level should be promoted and included in training of primary care providers.^15^

This study aimed to determine the prevalence of IPV amongst women attending antenatal care at a primary care facility in Outapi district, Namibia. In addition, the study aimed to assess the relationship between the different types of IPV (physical, sexual, emotional and economical) and to evaluate the presence of known risk factors.

## Methods

### Study design

This was a descriptive survey amongst pregnant women attending Outapi primary care clinic.

### Setting

The clinic is situated in the Outapi district near the major district hospital for the Omusati region in northern Namibia. It serves the local urban area and distant rural villages. According to 2011 census, Outapi town had an estimated population of 6437. The town comprises a range of white-collar and blue-collar workers, whereas the surrounding rural area consists mainly of village homesteads where most residents are unemployed. The clinic offers services such as antenatal care, postnatal care, family planning, mental health, child health and social work services. Because of the proximity of the clinic to Angola, some of the patients served are Angolans living along the Namibia–Angola border.

### Sample size and selection

The sample size calculated was based on the prevalence of 49.7% obtained in Botswana.^10^ Botswana shares cultural and demographic similarities and is in the same broad geographical setting as Namibia. To be within 5% of this prevalence, given 95% confidence intervals, a sample size of 385 was needed.

Women who had stayed in the catchment area of the clinic for at least six months were included regardless of nationality, race or ethnic group. Participants were selected consecutively as they attended the antenatal clinic. The research was conducted on antenatal care days over a five-week period. Pregnant women were seen in a private consultation room by two nurses who took a history and then performed an obstetric examination. During this consultation, the client was asked questions in order to ascertain whether she had experienced IPV or not. The clients who responded negatively were recorded as such together with the routine general socioeconomic information. The clients who responded affirmatively for experiencing IPV were invited to participate in the research. None of the women who responded affirmatively for experiencing IPV declined to participate in the study. Two consent forms were available, one in English and another in Oshiwambo, the local language. The women chose the language they were comfortable with and then made a voluntary decision to participate in the study.

### Data collection

Data were collected using a modified version of a validated questionnaire that was previously utilised in an antenatal clinic study in Soweto, South Africa.^1^ Modifications included having a small section on routine data collected from all women receiving antenatal care such as gestation, age, level of education, HIV status and marital status. The questions on assessing IPV were not changed; however, additional timeframes were added to include the previous 12 months, whilst pregnant and the number of times that abuse occurred. This questionnaire itself was a modified version of the WHO assessment questionnaire, which had been pretested in six countries, namely Bangladesh, Brazil, Namibia, Samoa, Thailand and the United Republic of Tanzania.^2^ A series of questions were asked to identify the forms of IPV as physical, emotional, financial and sexual abuse.

Two female nurses with good interpersonal skills, an interest in IPV and fluency in Oshiwambo administered the questionnaire, whilst they concurrently performed routine antenatal care. Piloting of the questionnaire did not suggest the need for any additional changes.

### Data analysis

Data were captured on an Excel sheet by a data clerk and checked for errors by the researcher. The data were analysed with the assistance of the Biostatistics Unit at Stellenbosch University. Determination of the relationship between demographic, clinical characteristics and IPV was done using the chi-square test. Otherwise, simple descriptive statistics were used to summarise the data.

### Ethical considerations

The study was approved by the Health Research Ethics Committee (S15/07/140) at Stellenbosch University and by the Ministry of Health and Social Services of Namibia. Confidentiality was of great importance to this study, and care was taken to ensure that there was no recording of names, address or other personal details of the participants. Participants who required more assistance after taking part in the study were further assisted by a social worker. The study was conducted as per the WHO’s ethical and safety recommendations for research on domestic violence against women.^16^ All participants were required to sign a written informed consent, which was either in English or the local language (Oshiwambo). Privacy and confidentiality were maintained at all times, and the questionnaire was administered as part of the routine consultation and examination in an enclosed room.

## Results

Altogether 386 pregnant women took part in the study, and their mean age was 27.5 years (standard deviation [SD] = 6.0). Table 1 shows the frequency distribution of the participants’ characteristics. Most women were unmarried, although may have been co-habiting with their partners, had only primary school education and were attending in their third trimester of pregnancy. Out of the 386 participants, 44 (11.4%) were teenagers.

**TABLE 1:** Characteristics of the participants (***N*** = 386).

Variable	*n* (%)
**Age (years)**	
14–19	44 (11.4)
20–25	126 (32.6)
26–31	103 (26.7)
32–37	72 (18.7)
38–43	38 (9.8)
44–49	3 (0.8)
**Gestation**	
First trimester	19 (4.9)
Second trimester	130 (33.7)
Third trimester	237 (61.4)
**Education**	
None	63 (16.3)
Primary	215 (55.7)
Secondary	103 (26.7)
Tertiary	5 (1.3)
**Marital status**	
Unmarried	335 (86.8)
Married	51 (13.2)
Separated	0 (0.0)
Divorced	0 (0.0)
**HIV status**	
Negative	335 (86.8)
Positive	51 (13.2)

### Prevalence of intimate partner violence

The reported lifetime prevalence of IPV was 39 (10.1%), the 12-month prevalence was 35 (9.1%) and the prevalence during pregnancy was 31 (8.0%). Table 2 shows the prevalence of different types of IPV and the mean number of times that abuse occurred. All women who disclosed their history of IPV completed the questionnaire. Emotional abuse was the commonest type of IPV amongst the participants overall as well as during pregnancy.

**TABLE 2:** Prevalence of intimate partner violence (*N* = 386).

Type of abuse	Ever occurred *n* (%)	Last 12 months *n* (%)	Whilst pregnant *n* (%)	Mean (SD)
Physical abuse	17 (4.4)	17 (4.4)	13 (3.4)	2.6 (1.5)
Emotional abuse	30 (7.8)	27 (7.0)	23 (6.0)	2.7 (1.3)
Financial abuse	23 (6.0)	20 (5.2)	20 (5.2)	3.2 (2.3)
Sexual abuse	11 (2.8)	9 (2.3)	6 (1.6)	3.2 (2.8)

### Forms of intimate partner violence

Within each category, different forms of IPV were assessed using specific questions. Table 3 shows the different forms of IPV. Failure to provide money to run the house or look after the children but having money for other things was the most common specific form of IPV, with 23 (6.0%) reporting it. Severe forms of physical abuse such as burning, threatening or using a gun were not reported by the participants.

**TABLE 3:** Prevalence of different forms of intimate partner violence.

Forms of intimate partner violence	Ever occurred *n* (%)	Last 12 months *n* (%)	Whilst pregnant *n* (%)	Mean number of times occurred
**Physical abuse**			
Has he ever pushed you or shoved you?	12 (3.1)	10 (2.6)	7 (1.8)	11
Has he ever slapped you or thrown something at you, which could hurt you?	14 (3.6)	12 (3.1)	9 (2.3)	12
Has he ever hit you with his fist or something else that could hurt you?	11 (2.8)	10 (2.6)	5 (1.3)	10
Has he ever kicked you, dragged you?	4 (1.0)	4 (1.0)	1 (0.3)	4
Has he ever beaten you up?	10 (2.6)	10 (2.6)	6 (1.6)	10
Has he ever strangled you?	3 (0.8)	2 (0.5)	2 (0.5)	3
Has he ever burnt you purpose?	0 (0.0)	0 (0.0)	0 (0.0)	0
Has he ever threatened to use or actually used a gun?	0 (0.0)	0 (0.0)	0 (0.0)	0
Has he ever threatened to use or actually used a knife or another weapon against you?	0 (0.0)	0 (0.0)	0 (0.0)	0
**Emotional abuse**			
Has he ever forced you to leave the place where you were living?	8 (2.1)	7 (1.8)	5 (1.3)	7
Has he ever forced your children to leave the place where you were living?	4 (1.0)	3 (0.8)	2 (0.5)	3
Has he ever insulted you or made you feel bad about yourself?	20 (5.2)	18 (4.7)	14 (3.6)	17
Has he ever belittled or humiliated you in front of other people?	13 (3.4)	9 (2.3)	7 (1.8)	10
Has he ever tried to prevent you from seeing family or friends?	7 (1.8)	7 (1.8)	5 (1.3)	7
Has he ever tried to prevent you from speaking with other men?	16 (4.1)	15 (3.9)	8 (2.1)	13
Has he ever boasted about or brought home girlfriends?	18 (4.7)	15 (3.9)	13 (3.4)	16
Has he ever done things to scare or intimidate you on purpose, for example by the way he looked at you, by shouting and smashing things?	6 (1.6)	3 (0.8)	3 (0.8)	2
Has he ever threatened to hurt you?	11 (2.8)	8 (2.1)	7 (1.8)	8
**Financial abuse**			
Has he ever failed to provide money to run the house or look after the children but had money for other things?	23 (6.0)	19 (4.9)	20 (5.2)	17
Has he ever taken your earnings?	3 (0.8)	2 (0.5)	2 (0.5)	3
Has he ever tried to prevent you from going to work, sell or make money in any other way?	2 (0.5)	2 (0.5)	1 (0.3)	2
Has he ever forced you to do some work that you didn’t like?	1 (0.3)	0 (0.0)	0 (0.0)	0
**Sexual abuse**			
Has he or any other partner ever physically forced you to have sex when you did not want to?	10 (2.6)	8 (2.1)	4 (1.0)	2
Have you ever had sex when you did not want to with your current boyfriend or husband, or any other partner, because you were afraid of what he might do?	7 (1.8)	6 (1.6)	4 (1.0)	6
Has your current boyfriend or husband, or any other partner, ever forced you to do something sexual that you found degrading or humiliating?	3 (0.8)	2 (0.5)	1 (0.3)	2

### Associated factors

There was no significant relationship between the prevalence of IPV and the level of education, gestation, HIV status or marital status. There was a significant association (*p* = 0.007) between IPV and older age with the mean age of those who had experienced IPV being 30.3 years (95% CI: 29.4–35.7) and those without an experience of IPV being 27.2 years (95% CI: 26.1–32.4).

## Discussion

In this study, 10.1% of pregnant women had experienced IPV during their lifetime, 9.1% in the last 12 months and 8.0% during the current pregnancy. Emotional abuse was the most common type of IPV followed by financial, physical and then sexual abuse. Older pregnant women were more likely to have experienced IPV.

The lifetime prevalence of IPV was within the range of 2.0%–13.5% found in 19 other countries from Africa, Latin America, Europe and Asia.^14^ The prevalence of physical abuse during pregnancy of 3.4% is similar to that obtained by the Namibian Demographic and Health Survey (DHS) for the Omusati region of 3.8%.^10^ This is consistent with the finding that this region is amongst the regions in Namibia with lower rates of IPV. Of note, however, is that in the Namibian DHS of 2013, only ‘physical abuse’ was assessed and documented in pregnancy. It remains therefore unknown how the prevalence of other types of IPV obtained in this study compares.

The rate of physical abuse during pregnancy in this study was much lower than the prevalence obtained in Windhoek, Namibia, of 6.1%.^2^ This difference could be because of a lower prevalence in rural areas or cultural differences in the study populations. It could also be attributed to the use of different methods. The Windhoek study was conducted through in-depth interviews and focus group discussions by well-trained female research assistants.^2^ It is possible that women were less likely to disclose IPV to a nursing sister at the local antenatal clinic, especially if they felt ashamed or had previously experienced unsupportive responses from health providers.^17^ Underreporting can be attributed to the silence that is because of the shame that women feel, their loyalty to their partners and the stigma around IPV as a private matter.^18^ Subconscious defences such as denial and rationalisation of their experiences as ‘normal’ could also have affected their responses. Noteworthy, too, was that sexual coercion by partners was probably underreported as many women believe that a male partner has a right to have sex with her whenever and however he wants it.^18^

In comparison with other African countries, the results are comparable to those obtained in Uganda and Malawi.^14,19^ In comparison, however, to data from neighbouring countries such as Zimbabwe, Botswana and South Africa, the prevalence was much lower.^11,12,13^ The contrast may point to important differences in the way men respect and relate to women in Namibia. More research is needed to compare male views of IPV and cultural practices which enhance or diminish male respect for women across these countries. A cross-sectional study in Zimbabwe, conducted in low-income urban facilities, obtained a prevalence of 61.3% during pregnancy.^12^ This huge difference may be because of factors such as political and socioeconomic instability, conflicting cultural, moral and religious backgrounds, as well as urban–rural divides.

By contrast, this study was conducted in one semi-rural facility. Similar studies in Botswana and Rwanda also had much higher IPV lifetime prevalence rates of 49.7% and 35.1%, respectively. The Botswana study was conducted in a semi-urban public hospital, whereas the Rwandan study was conducted in two rural settings.^10,20^ Namibia, therefore, may have a lower prevalence of IPV than other African countries, and further research could explore the reasons for this.

This study also assessed the different types of IPV experienced by pregnant women. Emotional abuse was the commonest type of abuse, which was mainly demonstrated by the partner insulting or otherwise making the woman feel bad about herself. This is in agreement with observations that during pregnancy non-physical forms of violence tend to increase.^12^ Significantly, ‘severe forms’ of physical abuse such as burning, threatening to use a gun, knife or another weapon were not observed in this study. This also implies cultural or societal norms that are less violent than in neighbouring countries.

The study found that there was an association of reported IPV with an increase in age. This is in contrast with the data obtained by the Namibian DHS of 2013 which highlighted that IPV was more likely to be reported by younger women.^9^ This study assessed four types of IPV in contrast to the Namibian DHS of 2013 which only looked at ‘physical abuse’ in pregnancy. It could be that the association of IPV and age varies according to the type of IPV. Whilst younger women may be experiencing more physical abuse, the same cannot be concluded for the other non-physical types of IPV. Further studies will need to be conducted to assess this association. Nonetheless, other studies have obtained similar data showing that respondents aged 30–39 years reported violence more commonly compared to younger women.^21^ The reason for this is unknown in Namibia, though studies elsewhere have suggested that it could be younger women gaining more power as a result of pursuing education, employment and economic independence.^19^ The exact reasons in the Namibian context may need further investigation. There was no association between HIV status and IPV, and a similar result was obtained in a study in Rwanda.^20^ Other studies, however, have found an association between HIV and IPV.^22^ The lack of association in this study could be attributed to the relatively small number of HIV-infected individuals.

The majority of participants were single (86.8%). This is not surprising as most Namibian women are involved in unmarried relationships.^23^ This study found no association between IPV and marital status and level of education. A national cross-sectional household survey in eight southern African countries also found no association between IPV and level of education or marital status.^21^ The Namibian DHS of 2013 however found that violence during pregnancy decreased with increasing education.^9^ This is in agreement with findings from studies amongst sub-Saharan women which also showed that higher levels of education were associated with lower prevalence of IPV.^24^ The absence of this association could be as a result of a low number of educated women who participated in this study (1% with tertiary level education).

### Limitations

Possible underreporting has already been discussed. The low level of education (none or primary) in the majority of women could also be a contributing factor. A Zambian study revealed that the average person who had not completed primary school was less likely to report a violent argument with a partner.^21^ The study also relied on women remembering IPV which could lead to recall bias. The frequency of abuse was most likely underreported as several women responded with statements such as ‘many times’ or ‘several times’, which could not be further quantified. Not all risk factors were studied as, for example, no information was collected on characteristics of the intimate partner or economic status of the women.

### Recommendations

Because of the significant mental and physical health risks for women and their children associated with IPV and the prevalence found in this study, health service providers should implement the WHO’s clinical guidelines for IPV amongst women attending antenatal care.^25,26^ A potential model of care was developed in South Africa and found to be feasible and useful.^27^ In addition, as endorsed at the World Health Assembly, it is vital that IPV be included as a key issue within clinical governance. Operational research, however, will need to be conducted to identify which model of care for IPV will best work for the Namibian health system. Qualitative research could explore societal and cultural reasons for the range in prevalence and nature of IPV across southern Africa.

## Conclusion

The lifetime prevalence of IPV amongst pregnant women in a primary care setting in a semi-rural area of Namibia was 10.1%, 9.1% during the last 12 months and 8.0% during pregnancy. The study found that all types of IPV occurred during pregnancy, with emotional abuse being the commonest category (a common form of abuse being the partner insulting or making the woman feel bad about herself), followed by financial abuse. Overall the commonest specific abusive behaviour was failing to provide money for her to run the house or look after the children despite him having money for his priorities. Marital status, HIV status, gestational age and level of education did not demonstrate any association with IPV, although older pregnant women were more likely to have experienced IPV.
